# Person-centred integrated primary care for refugees: a mixed-methods, stepped wedge design study to assess the impact

**DOI:** 10.1017/S1463423625000167

**Published:** 2025-02-26

**Authors:** Rabia Çinar, Mieke de Klein, José Renkens, Reinier Akkermans, Mursal Latify, Bart Walewijn, Maria van den Muijsenbergh, Tessa van Loenen

**Affiliations:** 1 Radboud University Medical Centre dep. Primary and Community care, Nijmegen, the Netherlands; 2 HAN University for applied sciences, Nijmegen, the Netherlands

**Keywords:** Child health professional, GP, mental health, primary care, psychological problems, refugee minor, social worker

## Abstract

**Aim:**

To assess the impact of a person-centred culturally sensitive approach in primary care on the recognition and discussion of mental distress in refugee youth.

**Background:**

Refugee minors are at risk for mental health problems. Timely recognition and treatment prevent deterioration. Primary care is the first point of contact where these problems could be discussed. However, primary care staff struggle to discuss mental health with refugees.

Guided by the needs of refugees and professionals we developed and implemented the Empowerment intervention, consisting of a training, guidance and interprofessional collaboration in four general practices in the Netherlands.

**Methods:**

This mixed-method study consisted of a quantitative cohort study and semi-structured interviews. The intervention was implemented in a stepped wedge design. Patient records of refugee youth and controls were analysed descriptively regarding number of contacts, mental health conversations, and diagnosis, before and after the start of the intervention.

Semi-structured interviews on experiences were held with refugee parents, general practitioners, primary care mental health nurses, and other participants in the local collaboration groups.

**Findings:**

A total of 152 refugees were included. Discussions about mental health were significantly less often held with refugees than with controls (16 versus 38 discussions/1000 patient-years) but increased substantially, and relatively more than in the control group, to 47 discussions/1000 patient-years (compared to 71 in the controls) after the implementation of the programme.

The intervention was much appreciated by all involved, and professionals in GP felt more able to provide person-centred culturally sensitive care.

**Conclusion:**

Person-centred culturally sensitive care in general practice, including an introductory meeting with refugees, in combination with interprofessional collaboration, indeed results in more discussions of mental health problems with refugee minors in general practice. Such an approach is assessed positively by all involved and is therefore recommended for broader implementation and assessment.

Box 1. Content of the Empowerment intervention

1. **Training**, developed in co-creation with GP doctors and mental health nurses and refugee parents.

Duration:

4 hours training in GP practice, with 3 × 1 hour online booster session after 3, 6, and 9 months to discuss experiences of refugee youth with distress problems in practice and questions/issues on how to provide person-centred culturally sensitive care.

Trainers:

GP experienced in person-centred culturally sensitive care together with refugee doctors and parents.

Content:

- Presentation of refugee trainer about daily life, stressors, healthcare experiences, and expectations of refugees
- Interactive presentation on mental distress in refugee youth, how to recognize this, how to discuss this, and what social support/specialist treatments are locally available
- Role play with refugee trainer on how to build trust and discuss psychosocial issues and how to involve a professional interpreter in consultations
- Exercise to get acquainted with the practice guidance and materials
- Discussion of cases from their own practices
- **2. Practice guidance**

A poster (see below) with reminders of important aspects of person-centred culturally sensitive care and items to be discussed during the introductory meeting with new refugee patients. For each practice, relevant telephone numbers and contact details were added for interpreter services, mental health care and youth care, and social support.

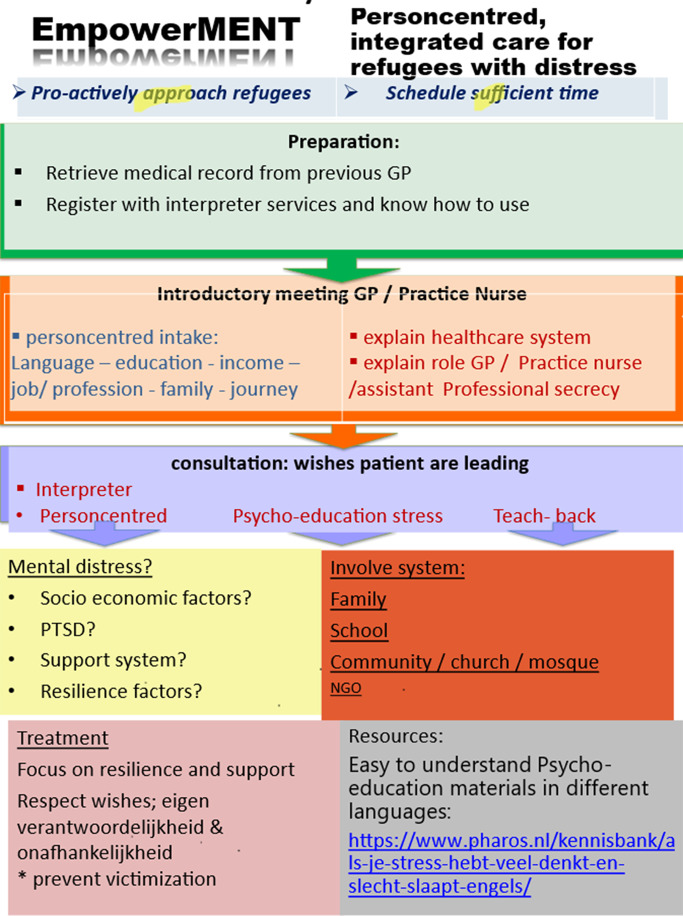

**3. Local interprofessional collaboration groups**

Around each participating GP a group was formed of GP/MHN, refugee representatives, and organizations in the field of support of refugee minors. These interprofessional groups met regularly to discuss activities to support refugee minors, to prevent or decrease mental distress.

## Introduction

Since 2015 some 56.9 thousand refugees settled in Dutch municipalities after being granted a refugee staying permit by the Dutch government (CBS (Dutch Central Bureau of Statistics), 2022). About 35% of the refugees are under the age of 18. After settling, they are enlisted in a general practice, as is the case with all Dutch people. In the Netherlands, the general practitioner (GP) is a gatekeeper to the healthcare system and the first point of contact for all health-related problems, including mental health problems for which also mental health practice nurses are available in GP to provide support and treatment. In case of severe mental health problems, patients are referred to specialist mental healthcare. Health insurance is mandatory for all Dutch citizens and covers costs for specialist care, including specialist mental healthcare; each year people have to pay the first costs (appr. 300 euros) themselves, except for general practice, which is free of charge. Youth care is also free of cost and is paid for by the municipality.

The traumatic experience of organized violence has been identified as a significant risk factor for mental health problems, like post-traumatic stress disorder (PTSD), depression, and anxiety disorders (Alisic *et al.*, 2014, Dangmann *et al.*, 2022). Prevalences of these problems vary widely between refugee groups and studies but are much higher than among non-refugee youngsters. For instance, the prevalence of PTSD among refugee children is estimated at 19–53%, compared to 16% in other children who experienced trauma (Dangmann *et al.*, 2022); depression is seen in 14% of refugee children worldwide compared to 3% in other children (Dangmann *et al.*, 2022). However, traumatic experiences are not the only nor major determinant of mental health of refugees. Other sources of chronic stress like insufficient household income and social exclusion have major long-term effects on health. They can lead to behavioural problems, sleeping disorders, eating problems, generalized pain, or bedwetting (Heptinstall *et al.*, 2004; Ehntholt & Yule, 2006; Bronstein & Montgomery, 2011; Pacione *et al.*, 2013; Dangmann *et al.*, 2022). As such, the well-being of children is related to that of their parents or guardians (Summerfield, 2000; Fazel *et al.*, 2012; Hirani *et al.*, 2016). Mental distress and mental health problems in refugee minors therefore are relevant for all health professionals and GPs in particular. GPs could have played a key role in the recognition of these problems in refugee minors. Yet there are indications of underdiagnosis of mental health problems in refugees and particularly refugee minors (Lamkaddem *et al.*, 2013; Dagevos *et al.*, 2018; Hodes & Vostanis, 2019). It seems more difficult to recognize mental distress and mental health problems across language and cultural differences, especially in groups that are not used to talking about these problems.

Therefore, the two-year Empowerment programme was developed and implemented in four general practices, to increase the awareness and skills of GPs to recognize, discuss, and attend to mental distress and health problems in refugee minors. In this mixed-methods study, we evaluated this programme and aimed to answer the following research questions:

1. Does a programme aimed at improving culturally sensitive person-centred integrated care and interprofessional collaboration in general practice increase the recognition, discussion, and guidance of mental distress and health problems in refugee youth?

We hypothesized first that before the implementation of the programme the number of general practice consultations with refugee minors in which mental health is being discussed would be lower than in other minors, and second that this number would be increased after the implementation of the programme.

1. How is the programme experienced by the GP staff and others involved in the programme?

## Methods

### Setting

From September 2019 until September 2021, four general practices in four different municipalities in South-Eastern Netherlands engaged in the Empowerment project (Radboud University, 2019). This project aimed to improve the recognition, discussion, and guidance of mental distress in refugee children. Based on literature and interviews with refugees, doctors, and mental healthcare nurses (MHNs) in general practice, as well as other professionals involved in the support of refugee children, we developed training and guidance for culturally sensitive person-centred care for GP staff (see box 1 for the content of the Empowerment programme). After the training, with the help of the guidance, the GPs and MHNs started their part of the intervention that existed of an extensive introductory meeting with each refugee family in their practice. In the introductory meeting with the refugee parents, sometimes in the presence of their children, attention was paid to medical problems, but also to the family composition and history, and their social and financial circumstances.

Besides, in the four participating municipalities meetings were organized involving the GP/MHN, refugee representatives and organizations in the field of support of refugee minors. The goal of these meetings was to strengthen interprofessional collaboration and psychosocial support tailored to the refugees’ needs.

The implementation of the programme was hampered by multiple lockdowns due to the SARS-COVID-19 pandemic, which also burdened general practices.

### Design

This mixed-method study consisted of a quantitative cohort study to answer research question one and qualitative semi-structured interviews to answer research question two.

For this report, we used a checklist specifically for mixed-methods studies (the Fetters, M. D., & Molina-Azorin, J. F. (2019). In this section, we first describe the methods applied for the quantitative cohort study and then those applied for the qualitative interviews

#### Cohort study

##### Design

We studied patient records in four general practices. We compared the number of GP consultations in which mental health was discussed in refugee minors with the number of these consultations in other minors before (from 01-09-2014 to 01-09-2019) and after the implementation of the Empowerment programme (from 1-1-2020 to 1-9-2021). We chose to study a five-year period prior to the implementation of our programme, as we wanted a substantial number of consultations to assess whether or not the number of discussions on mental health was lower in refugee youth (our first hypothesis). After the implementation of the intervention, our possibilities for evaluation were limited to two years. However, we adjusted our results to patient-years, to be able to compare both periods.

The implementation of the programme (01-09-2019 till 01-09-2021) was performed as a stepped wedge design study. In a stepped wedge design, every cluster starts with a control period. Then, each cluster starts with the intervention (in this case the Empowerment programme) at a different time. At the end of the stepped wedge design study, all clusters had implemented the intervention (Zhan *et al.*, 2014).

**Figure 1.**
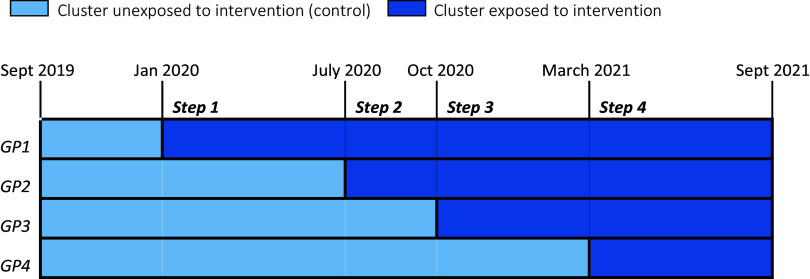
Stepped wedge design of Empowerment intervention.

This resulted in the following five steps (see Figure 1). Due to the COVID-19 pandemic; the start of practices 2, 3, and 4 was delayed with a shorter post-intervention period as a result.

#### Study population

The study population consisted of all minor refugee patients, that is, children of parents who both came to the Netherlands as refugees less than 10 years ago and registered with the participating four GP practices on 1 September 2014. Their data were manually selected from the patient records, based on surname/country of birth. In doubt, the researcher asked the GP whether the patient indeed was a refugee.

In this case-control design, we matched each refugee minor in the participating practice to the first control minor of the same gender and age group in that practice, of whom at least one parent was born in the Netherlands. After identification, the patient records were anonymized.

#### Data collection cohort study

The following information was extracted from the patient records:

Age and gender, country of origin of parents, number of consultations between 1-9-2014 and 1-9-2021, divided into the period before and after the start of the Empowerment programme in that particular practice; diagnoses coded according to the ICPC (International Classification of Primary Care coding); specific ICPC codes of the P category (referring to psychosocial problems/mental distress); and mentioning of discussion of mental health issues and referrals to mental healthcare or social care.

#### Data analysis cohort study

The data from the first five years (2014–2019) were descriptively analysed before the start of the study to answer our first hypothesis. Mean and standard deviation (std) or median and interquartile range for continuous characteristics and number and percentages for categorical characteristics were determined. The difference in the number of consults and diagnoses between the refugee group and the control group was tested by the incidence rate ratio (IRR). For the second hypothesis, we used a mixed-effect logistic model with practice as a random factor and group (intervention/control period) and step as fixed effects in the model. The difference in percentages of consultations and discussions about mental health before and after the start of the Empowerment programme was expressed as an odds ratio (OR) with 95% confidence interval.

A value of p < 0.05 was considered statistically significant for all analyses, based on two-sided testing. Analyses were performed using the Statistical Package for Social Sciences (SPSS, IBM Corp., Armonk, NY) version 25.

#### Qualitative semi-structured interviews

##### Design

In order to develop an intervention tailored to the needs of refugees and GPs, at the beginning of the study, we interviewed 15 refugee parents, 6 GPs, and 4 MHNs to elicit their experiences with refugee children and mental distress or mental health problems.

#### Study population for the qualitative semi-structured interviews

Refugee participants were recruited from the network of the authors (ML, JR, MvdM, MdK) through purposive sampling, striving for diversity regarding gender, age, educational background, and country of origin. Before deciding whether to participate, all participants received elaborate information about the goals, methods, and procedures of the study.

The four participating general practices, with a total of seven doctors and four MHNs, were also recruited through the network of the authors (BW and MvdM). Before the start of the study, all but one (one doctor was not available at that time) were interviewed.

The participants in the local interprofessional collaboration groups were recruited by the local practices.

For our interviews about the experiences with the Empowerment programme after the intervention, we recruited a convenience sample of in total 12 participants (five GPs and two MHNs, four other healthcare or social workers and one refugee representative who all had participated in the local interprofessional collaboration groups).

#### Data collection and analysis of the qualitative interviews

The topic guide for the interviews before the start of the intervention, based on literature and expert opinion, contained questions about experiences with and knowledge of mental distress and mental health problems, health-seeking behaviour of refugees, barriers, and facilitators in accessing and providing care and experiences with GP care for refugees.

The topic guide for the interviews on experiences with the programme, also based on literature and expert opinion and on the pre-intervention interviews, contained questions about the content and provision of the training, the guidance, the practicalities of the implementation of the guidance in practice, the self-assessed ability to address the needs of refugee patients and possible improvement in this after the implementation of the programme, and the experiences with interprofessional collaboration before and after the implementation of the programme.

The interviews were performed by several researchers (authors RÇ, BW, JR, and MdK). The interviews were recorded and transcribed ad verbatim using F4 software. Data of respondents will be stored for 15 years at the research location of the Radboud University Medical Centre.

All transcripts were carefully read by the researchers and inductively coded, using ATLAS.ti software (version 8.4.20). To secure data validity, all interviews were double-coded by at least two researchers and differences were discussed until agreement was reached. The codes were merged into overarching categories and themes.

## Results

### Cohort study

#### Characteristics of the study population

In total, 152 refugee minors from 72 families were enlisted in the four participating general practices, with in total 16,394 patients on their practice list: GP1 (total practice list 7108 patients): 65 refugees (28 families); GP2 (total practice list 4265 patients): 49 refugees (25 families); GP3 (total practice list 2849 patients): 34 refugees (17 families); and GP4 (total practice list 2172 patients): 4 refugees (2 families).

Of these refugee minors, 57% were male and 43% female. The refugees originated from 21 different countries. Most parents came from Syria (52.0%) or Eritrea (8.6%). Nearly a quarter (22.7 %) of the refugee minors were born after their parents arrived in the Netherlands.

In the five years prior to the intervention (01-09-2014 to 01-09-2019), there were in total of 1023 consultations with 152 refugee minors (1.4 per minor per year), compared to 1677 consultations in the control group (2.2 per minor per year).

During the two-year intervention period, 24 refugee minors and 19 control minors moved so their records could not be longer included in the evaluation. No new refugee minors were registered in the participating practices. After the implementation of the intervention, the number of consultations with refugee minors increased to a total of 604 consultations with 116 refugee minors (out of the total of 128 enlisted) (2.6 per minor per year), compared to 561 consultations in 117 controls (out of the 133 enlisted) (2.4 per minor per year).

#### Number of discussions about mental health and P-diagnoses before and after the intervention

In the five years before the start of the intervention, significantly fewer discussions of mental health were registered in the refugee group: 16 discussions per 1000 patient-years, compared to 38 discussions per 1000 patient-years in the control group (IRR 2.89 [95% CI 1.43, 6.21], p = 0.0046) (see Table 1). In this period before the start of the intervention, also significantly less often a P-diagnosis (psychological distress or problem) was registered in the refugee children: 70 diagnoses/1000 patient-years compared to 128 diagnoses/1000 patient-years in the controls (IRR 1.83 [95% CI 1.30, 2.61], p = 0.0003) (see Table 2). This confirmed our first hypothesis.

**Table 1.** Number of patients with whom mental health is discussed (and as %* of patients and per 1000 patient-years) before and after the implementation of the programme (refugee minors compared with their controls)

	5-year period before the implementation				0.7–2 years after the implementation			
	Refugee minors		Control minors		Refugee minors		Control minors	
	N	Number of refugee minors with whom mental health is discussed (and as % of patients)	N	Number of control minors with whom mental health is discussed (and as % of patients)	N	Number of refugee minors with whom mental health is discussed (and as % of patients)	N	Number of control minors with whom mental health is discussed (and as % of patients)
GP1 7108 patients on practice list	65	3 (5%)	65	16 (25%)	49 20 months of intervention	5 (10%)	54	4 (7%)
GP2 4265 patients on practice list)	49	1(2%)	49	9 (18%)	44 14 months of intervention	5 (11%)	44	7 (16%)
GP3 (2849 patients on practice list)	34	6 (18%)	34	4 (12%)	31 11 months of intervention	2 (7%)	32	8 (25%)
GP4 (2172 patients on practice list)	4	2 (50%)	4	0 (0%)	4 7 months of intervention	0 (0%)	3	0 (0%)
**Total** (16394 patients on practice list)	**152**	**12 (8%)** 16 discussions per 1000 patient-years	**152**	**29 (19%)** 38 discussions per 1000 patient-years	**128**	**12 (9%)** 47 discussions per 1000 patient-years	**133**	**19 (14%)** 71 discussions per 1000 patient-years

* Given the small total number, percentages are rounded off to whole numbers.

**Table 2.** Number of patients with a P-diagnosis (and as % of patients and per 1000 patient-years) before and after the implementation of the Empowerment programme (refugee minors compared with their controls)

	Before the implementation						After the implementation					
	Refugee minors			Control minors			Refugee minors			Control minors		
	N	Number of refugee minors with a P-diagnosis (% of the total group of refugee minors)	Number of p-diagnoses (p-diagnoses/1000 patient-years)	N	Number of control minors with a P-diagnosis (% of total group of refugee minors)	Number of p-diagnoses (p-diagnoses/1000 patient-years)	N	Number of refugee minors with a P-diagnosis (% of the total group of refugee minors)	Number of p-diagnoses (p-diagnoses/1000 patient-years)	N	Number of control minors with a P-diagnosis (% of total group of refugee minors)	Number of p-diagnoses (p-diagnoses/1000 patient-years)
GP1	65	19 (29%)	28 (86)	65	33 (51%)	64 (197)	49	8 (16%)	11 (112)	54	9 (17%)	16 (148)
GP2	49	7 (14%)	7 (29)	49	13 (27%)	14 (57)	44	6 (14%)	8 (91)	44	11 (25%)	25 (284)
GP3	34	12 (35%)	15 (88)	34	12 (35%)	19 (112)	31	7 (23%)	9 (145)	32	7 (22%)	17 (266)
GP4	4	3 (75%)	3 (150)	4	0 (0%)	0 (0)	4	1 (25%)	1 (125)	3	0 (0%)	0 (0)
**Total**	**152**	**41 (27%)**	**53 (70)**	**152**	**58 (38%)**	**97 (128)**	**128**	**22 (17%)**	**29 (113)**	**133**	**27 (20%)**	**58 (218)**

In the two years after the start of the Empowerment programme, the percentage of discussions about mental health within the refugee minor groups increased from 16 to 47 discussions per 1000 patient-years (from 8% of all children in 5 years to 9 % in 2 years) (OR = 1.21 [95% CI 0.52, 2.79], p = 0.66), although it also increased in the control group from 38 to 71 discussions per 1000 patient-years (but in that group, the percentage of children with whom mental health was discussed decreased from 19% to 14%) (OR = 0.71 [95% CI 0.38, 1.33] p = 0.28) (see Table 1). During this intervention period, also the number of refugee children who received a P (psychological) diagnoses increased: 22 of the 128 refugees received in total 29 P-diagnoses (115 diagnoses/1000 patient-years), compared to a still higher number of controls: 27 of the 133 controls received a total of 58 P-diagnoses (216 diagnoses/1000 patient-years) (IRR 1.88 [95% CI 1.18, 3.05], p = 0.0046) (see Table 2 and Supplementary Tables S1 and S2). So compared with the period before the intervention, there is an increase in the number of mental health discussions and of P-diagnoses in the refugee group, relatively more than in the control group. This is in concordance with our second hypothesis.

### Experiences of refugees

In order to develop an intervention tailored to the needs of refugees, we interviewed 21 refugee parents (see Table 3) from eight different countries about their experiences with mental distress in their children and with healthcare in the Netherlands, in particular their experiences and wishes regarding their GP.

**Table 3.** Number and gender of interviewees before and after the implementation of the intervention

	Before the implementation	After the implementation	Gender male	Gender female
Refugee adults	21 (5 couples)	1	8	14
GP	6	5 (out of the 6)	2	4 (3)
MHN	4	2 (out of the 4)	1 (0)	3 (2)
Social and community workers	0	4	1	3

These interviews showed that refugees and their children experience a lot of mental distress related to the traumas and difficulties they and their parents have experienced in their country of origin as well as now in the Netherlands. They initially seek support and help from family or religion or by engaging in distracting activities and only as a last resort do they turn for help to their GP. They experience barriers in accessing the GP practice and in discussing psychological problems with their GP. The most important barriers are the lack of an interpreter, the business-like approach of the GP, and the limited time available. Refugee parents also experience shame in discussing psychological problems with outsiders.

*‘Look, I have not had an easy husband, he has been in prison and experienced war in our country of origin, his father and brother were murdered in front of his eyes. So my husband was confused, traumatized and unstable. And my children were very vulnerable to that. They could easily go the wrong way. As a mother you then have to be extremely strong’. (Afghan woman)*

*‘We noticed here in children who are 12 or 13 years old that they are now stuck between the country of origin and the Netherlands. If they behave like the people in the country of origin, then that is not accepted by the Dutch people. If they act like the Dutch, they will not be accepted by their parents and the community from their country of origin. So they are stuck in between. They don’t know what to do, they feel lost on the road’. (Eritrean man)*

*‘If the general practitioner wants to improve something for foreigners, the first thing is the language. I have been here for more than four years, but I still do not dare to go to the general practitioner immediately, because I do not know how to explain my complaints in ten minutes, that is not enough time’. (Syrian woman)*

*‘Our culture is closed, we do not want our problems to be known and talk about it. If an adult is stressed, he will not say so and eventually it will get worse. So the culture prevents it’. (Eritrean man)*

### Experiences of GP staff and other stakeholders

The interviews with six GPs and four MHNs from the participating practices (see Table 3) before the start of the Empowerment programme showed that they were aware mental distress and mental health problems are common in refugee children. However, they experience barriers in discussing these problems. The most important barriers mentioned by professionals were refugees’ limited understanding and mistrust of the Dutch healthcare (system), language barriers, the limited time of professionals, expected cultural differences, and the fact that a physical complaint is often the reason for consultation although the origin might be mental distress.

*‘You just feel powerless. you feel there are a lot of issues in the life of this refugee patient, but it is difficult to ask about, given the language barrier, and then this cultural thing you do not know about’. (MHN1)*

*‘Patients from other countries, I see, they are more often body oriented, do they have more physical complaints, and then you ask about some psychosocial topics and there are many problems, so you think ‘of course you are not sleeping well’. (GP3)*

Our interviews with GPs and MHNs after the implementation of the programme showed that all three elements of the intervention were equally important: the training was a necessary start, as it raised awareness as well as provided the skills for culturally sensitive communication about mental distress; the booster sessions helped them to solve difficulties they encountered in providing care for refugees with mental distress; and the practice guidance was experienced as equally important as it was easy to consult (it was in the form of a poster) and contained telephone numbers of interpreter services and of locally available support and mental healthcare services. It also contained detailed guidance for the introductory meeting that was advised to build trust and get to know the refugee family, but due to time restraints, these introductory meetings were not held with all refugees. However, when they were held, they were experienced as very effective. Both GPs and MHNs felt that this introductory meeting helped to develop mutual understanding and a relationship of trust.

After the intervention, the GP practices started to use the telephone interpreter services more often, and to their appreciation, but still, consultations remain complicated due to cultural and language barriers.

*‘You could say I was more aware of it. After that training of yours I think oh yes good to think about it and think about it for a while. That’s the most important thing to me’. (GP4)*

*‘And I think there is a lot of added value in speaking to those people yourself and just getting to know your own patients much better. That you just build a bond a little faster and get to know your patients a little faster. That’s what got me the most’. (GP1)*

*‘The guidance we received for the extensive refugee history, was also very nice, as we use them as a stepping stone for an introductory meeting’. (GP1)*

*‘I have become more aware of refugees in our practice. To put yourself in their shoes and how they can experience things. That did help me’. (MHN)*

*‘Well, I actually think I got just a new perspective, a different view and more attention for refugees in practice. Yes, I think that’s the most important thing. Also tools and practical skills that you can use in practice’. (GP1)*

The interprofessional collaboration groups were also experienced as helpful. The participants felt that their collaboration with other organizations and professionals had improved because they now knew each other and could easily find each other which improved their communication and contributed to a shared approach with clear division of tasks.

*‘By knowing people, by having a network, you indeed have many possibilities…. apparently so many more people are actually reachable than what you think in advance if you don’t visit each other’. (Community worker)*

*‘Especially, being in a group with the GP for the first time, I found was very different’. (Social worker)*

## Discussion

### Main results

As we hypothesized, mental health was discussed significantly less often with refugee minors than with minors from the control group. After the implementation of our intervention to improve culturally sensitive person-centred care, the number of mental health discussions and of mental health diagnoses in refugee minors increased substantially.

Interviews with refugees as well as GP staff before the start of the programme indicated that stress is a very common problem, and there are barriers to discuss this. The most important barriers mentioned by both parties were the lack of an interpreter and the limited time available. Refugee parents also mentioned the business-like approach of the GP and shame, whereas GP staff added as barriers.

Refugees’ mistrust of the Dutch healthcare and expected cultural differences.

The Empowerment programme was positively assessed by all professionals involved. All three elements of the intervention in the GP were experienced as equally important: the training and booster sessions, the practice guidance, and introductory meetings, although due to time restraints, these introductory meetings were not held with all refugees. However, when they were held, they were experienced as very effective.

### Comparison with literature

In line with our findings also other studies indicate that both refugees and other migrants and healthcare professionals experience barriers in establishing the necessary trust to discuss sensitive topics like mental distress, due to language and cultural differences as well as time constraints (Fazel *et al.*, 2012; Suphanchaimat *et al.*, 2015; Loenen *et al*., 2018; Zendedel *et al.*, 2018; Hodes & Vostanis, 2019; Iliadou *et al.*, 2019); Fair *et al.*, 2021; Jager *et al.*, 2021). In addition, refugees experience various barriers to accessing health care (Loenen *et al*., 2018; Van der Boor & White, 2020; Hodes & Vostanis, 2019). On top of this, we know from other studies that when experiencing stress, they tend to seek out other forms of support first, before contacting professional help (Teunissen *et al.*, 2014; Renkens *et al.*, 2022). The physical presentation of stress-related complaints is often outpointed by GPs as a challenge in communication with immigrant groups (Hjörleifsson, Hammer, & Díaz, 2017). A language barrier is acknowledged as a major challenge, especially for psychosocial consultations (Oehri *et al.*, 2023) A professional interpreter must be involved in consultations with refugees, as this is known to be the only way possible mental health issues will be discussed (Krystallidou *et al.*, 2020; Zendedel *et al.*, 2023).

Not only do the skills and attitudes of professionals have to be improved, but also structural barriers have to be removed like limitations put in place by the government, health insurance, or others regarding the availability of interpreter services and sufficient time for professionals (McFarlane, 2021).

We did not find any studies where mental health care for refugees in general practice was compared with this care for other groups. However, we know GPs discuss less often other sensitive topics, like sexual and reproductive care, with refugees and other migrants than with non-migrant patients (Raben & van den Muijsenbergh, 2018).

A review showed that most interventions to improve primary care for refugees focus on upskilling doctors, with a paucity of research exploring the involvement of other healthcare members (Iqbal *et al.*, 2022). Our intervention involved other healthcare professionals as well as an interprofessional team, as was recommended in the review (Iqbal *et al.*, 2022). The importance of collaboration with a local interprofessional team was also pointed out by other researchers, as the intense health needs of refugees require an integrated community-based primary healthcare approach (McMurray *et al.*, 2014)

In other fields of primary care – midwifery care (Fair *et al.*, 2021) and dietetic care (Jager *et al.*, 2023) – training in culturally sensitive person-centred care and in particular in cross-cultural communication was also evaluated as positive. However, to prove the positive effect of training in cultural competency on patient outcomes (e.g. in the mental health of refugee minors), more systematic and large-scale development and evaluation of such training are required, including assessment of real-life behaviour of professionals and the experiences of patients (Jager *et al.*, 2023).

To our knowledge, the other elements of our intervention (the practice guidance as well as the introductory meeting with new refugee patients) have never been studied before. However, GP staff frequently mention difficulties in finding practical information on interpreter services or support organizations during their consultations (Papadakaki *et al.*, 2017; Teunissen *et al.*, 2017). Our guidance was designed to support professionals in this, and it was experienced as helpful.

The introductory meetings were aimed at increasing trust, which also in other studies is proven to be crucial for effective communication (Van den Muijsenbergh *et al.*, 2013).

A person-centred culturally sensitive approach in general has proven to create more trust in the healthcare professional and thus is likely to improve the discussion about sensitive issues with migrants as well the effectiveness of care (Betancourt, 2006; Renzaho *et al.*, 2013; Seeleman, 2014; Ahmed *et al.*, 2022; Ahmed *et al.*, 2023).

In order to provide culturally sensitive person-centred care, GPs will need sufficient time, as is pointed out by WHO (WHO & Unicef, 2020). Therefore, we are pleased that Dutch health insurers will enable GPs to spend more time on their patients from 2024 (LHV, 2023).

### Strengths and limitations

The stepped wedge design that is used is complex, but it is a strong design because the participants are both control and intervention (Zhan, 2014). In a stepped wedge design, the sample size could be smaller than in a typical cluster trial. We had a sample size of N = 152, which is a strength for the validity of this study. A disadvantage of the stepped wedge design is that it has a longer trial duration (3–5 years). So, the limited time (two years) we had for this study may be a limitation for the validity, especially as the implementation of the intervention programme was hampered by the COVID-19 pandemic and its restrictions on meetings such as training sessions.

The COVID-19 pandemic also made the GPs busier but at the same time resulted in fewer consultations. Our finding that despite this, the number of discussions on mental health increased in the refugee population is an indication that our intervention supported GPs and MHNs in improving care for this group. On the other hand, as mental distress increased during the COVID-19 pandemic, specifically in refugees (Padilla *et al.*, 2021), the increase in discussions on mental health after our intervention could also, at least partially, be caused by an increase of mental distress.

Keeping patient records is time-consuming for GPs. During the data collection, we saw that patient records were not always complete. This is also reflected in the variable ‘unknown’ in the results of, for example, the diagnosis. So, mental health problems may be discussed by the GP but remain invisible in the patient records.

### Recommendations

For future research:

In this study, we focus on GPs discussing mental health problems in refugee minors, as this is the starting point for treatment or guidance. Our intervention seems to improve the number of these discussions; however, of course, the ultimate aim of our intervention is to improve the well-being of refugee youth and their families. Further research is needed to see whether or not this would be the case.

For GP practices:

Enable the provision of person-centred culturally sensitive care by:

- Organizing access to (telephone) interpreter services
- Allowing time for introductory meetings, prolonged consultations, and interprofessional collaboration meetings as well as post-graduate education on person-centred culturally sensitive care
- Providing easy-to-understand multilingual information on practice organization and on health promotion issues
- Involving migrants in the assessment and development of practice organization and procedures (O’Reilly-deBrún 2017, MacFarlane *et al.*, 2021)

For GPs and mental health practice nurses:

- Invest in trust by getting to know your patients by arranging an extensive introductory meeting with patients when registering with the practice. This meeting should address not only physical but also psychosocial aspects, language, and living circumstances. Besides it should explain how the healthcare systems work and how all staff are bound to acknowledge patient confidentiality.
- Involve professional interpreters in your consultations.
- Get to know and work together with other organizations and services that can support refugees (youth).
- Be aware of possible shame or stigma surrounding mental health issues, but do ask about mental distress by normalizing it, explaining how the body and mind react to stressors.

## Conclusion

Person-centred culturally sensitive care in general practices, including an introductory meeting with refugees in combination with interprofessional collaboration regarding the mental health of refugee minors indeed results in more discussions of mental health problems with refugee minors in general practices. Such an approach is assessed positively by all involved and is therefore recommended for all general practices.

## Supporting information

Çinar et al. supplementary material 1 Çinar et al. supplementary material

Çinar et al. supplementary material 2 Çinar et al. supplementary material

## Supplementary material

The supplementary material for this article can be found at https://doi.org/10.1017/S1463423625000167.
