# Supplementary material for: Person-centred integrated primary care for refugees: a mixed-methods, stepped wedge design study to assess the impact
Source: Prim Health Care Res Dev. 2025 Feb 26;26:e17. doi: 10.1017/S1463423625000167 (PMC11883791; doi:10.1017/S1463423625000167)
Supplement: Çinar et al. supplementary material 1 — Çinar et al. supplementary material [file S1463423625000167sup001.docx]

Table S1: Total number of patients with a P-diagnosis (and as %* of patients) in the 5 years before the start of the Empowerment program (2014 – 2019) (refugee minors compared with their controls)

|  | GP1 (7108 patients on practice list) | | GP2 (4265 patients on practice list) | | GP3 (2849 patients on practice list) | | GP4 (2172 patients on practice list) | | Total of 16394 patients | |
| --- | --- | --- | --- | --- | --- | --- | --- | --- | --- | --- |
|  | Number of refugee minors with P diagnosis (N=65) | Number of controls with a P diagnosis (N=65) | Number of refugee minors with P diagnosis (N=49) | Number of controls with a P diagnosis (N=49) | Number of refugee minors with P diagnosis (N=34) | Number of controls with a P diagnosis (N=34) | Number of refugee minors with P diagnosis (N=4) | Number of controls with a P diagnosis (N=4) | Number of refugee minors with P diagnosis (N=152) | Number of controls with a P diagnosis (N=152) |
| **P01** Anxiety | 0 (0%) | 0 (0%) | 0 (0%) | 1 (2%) | 2 (6%) | 1 (3%) | 0 (0%) | 0 (0%) | 2 (1%) | 2 (1%) |
| **P02** Crisis, short stress reaction | 0 (0%) | 0 (0%) | 0 (0%) | 1 (2%) | 0 (0%) | 0 (0%) | 0 (0%) | 0 (0%) | 0 (0%) | 1 (1%) |
| **P02.01** PTSD | 0 (0%) | 0 (0%) | 0 (0%) | 0 (0%) | 1 (3%) | 0 (0%) | 0 (0%) | 0 (0%) | 1 (0,7%) | 0 (0%) |
| **P03** Depressed, feeling down | 0 (0%) | 2 (3%) | 0 (0%) | 1 (2%) | 0 (0%) | 1 (3%) | 0 (0%) | 0 (0%) | 0 (0%) | 4 (3 %) |
| **P06** Sleeplessness/ | 0 (0%) | 5 (8%) | 1 (2%) | 1 (2%) | 0 (0%) | 1 (3%) | 1 (25%) | 0 (0%) | 2 (1%) | 7 (5%) |
| **P12** Enuresis | 5 (8%) | 2 (3%) | 0 (0%) | 0 (0%) | 0 (0%) | 1 (3%) | 0 (0%) | 0 (0%) | 5 (3%) | 3 (2%) |
| **P20** Memory/ concentration/ impairment | 0 (0%) | 0 (0%) | 0 (0%) | 1 (2%) | 0 (0%) | 1 (3%) | 0 (0%) | 0 (0%) | 0 (0%) | 2 (1%) |
| **P21** Hyperactivity/ hyperkinetic | 0 (0%) | 8 (12%) | 0 (0%) | 0 (0%) | 0 (0%) | 1 (3%) | 0 (0%) | 0 (0%) | 0 (0%) | 9 (6%) |
| **P22** Other worries behaviour child | 1 (2%) | 4 (6%) | 0 (0%) | 3 (6%) | 0 (0%) | 2 (6%) | 0 (0%) | 0 (0%) | 1 (1%) | 9 (6%) |
| **P24** learning problem | 0 (0%) | 1 (2%) | 1 (2%) | 1 (2%) | 0 (0%) | 1 (3%) | 1 (25%) | 0 (0%) | 2 (1%) | 3 (2%) |
| **P24.03** Motoric development disorder | 0 (0%) | 0 (0%) | 0 (0%) | 0 (0%) | 1 (3%) | 0 (0%) | 0 (0%) | 0 (0%) | 1 (1%) | 0 (0%) |
| **P24.2** Language/ speech deficiency** | 13(20%) | 7 (11%) | 5 (10%) | 1 (2%) | 5 (15%) | 2 (6%) | 1 (25%) | 0 (0%) | 24 (16%) | 10 (7%) |
| **P74** Anxiety disorder | 0 (0%) | 0 (0%) | 0 (0%) | 0 (0%) | 1 (3%) | 0 (0%) | 0 (0%) | 0 (0%) | 1 (1%) | 0 (0%) |
| **P79** Other neurosis | 0 (0%) | 0 (0%) | 0 (0%) | 1 (2%) | 0 (0%) | 0 (0%) | 0 (0%) | 0 (0%) | 0 (0%) | 1 (1%) |
| **P85** Mental retardation | 0 (0%) | 0 (0%) | 0 (0%) | 0 (0%) | 1 (3%) | 1 (3%) | 0 (0%) | 0 (0%) | 1 (1%) | 1 (1%) |
| **P99** Autism spectrum disorder | 0 (0%) | 4 (6%) | 0 (0%) | 2 (4%) | 1 (3%) | 0 (0%) | 0 (0%) | 0 (0%) | 1 (1%) | 6 (4%) |
| **Total***** | 19 (29%) | 33 (50%) | 7 (14%) | 13 (26%) | 12 (35%) | 12 (35%) | 3 (75%) | 0 (0%) | 41 (27%) | 58 (38%) |

**Given the small numbers, percentages are rounded off*

*** In contrast to the control minors, the refugee minors were primarily diagnosed with language/speech deficiency due to their lack of proficiency in the Dutch language, rather than factors such as stuttering.*

**** Some patients received more than one P-diagnosis*
