# Supplementary material for: Person-centred integrated primary care for refugees: a mixed-methods, stepped wedge design study to assess the impact
Source: Prim Health Care Res Dev. 2025 Feb 26;26:e17. doi: 10.1017/S1463423625000167 (PMC11883791; doi:10.1017/S1463423625000167)
Supplement: Çinar et al. supplementary material 2 — Çinar et al. supplementary material [file S1463423625000167sup002.docx]

Table S2: Number of patients per P-diagnosis (and as %* of patients) during the max 2 year period after implementation of the Empowerment program, (2019 – 2021) (refugee minors compared with their controls)

|  | GP1 | | GP2 | | GP3 | | GP4 | | Total | |
| --- | --- | --- | --- | --- | --- | --- | --- | --- | --- | --- |
|  | Number of refugee minors with P diagnosis (N=49) | Number of controls with a P diagnosis (N=54) | Number of refugee minors with P diagnosis (N=44) | Number of controls with a P diagnosis (N=44) | Number of refugee minors with P diagnosis (N=31) | Number of controls with a P diagnosis (N=32) | Number of refugee minors with P diagnosis (N=4) | Number of controls with a P diagnosis (N=3) | Number of refugee minors with P diagnosis (N=128) | Number of controls with a P diagnosis (N=133) |
| **P01** Anxiety | 0 (0%) | 1 (2%) | 1 (2%) | 0 (0%) | 1 (3%) | 0 (0%) | 0 (0%) | 0 (0%) | 2 (2%) | 1 (1%) |
| **P04** Irritable, anger | 0 (0%) | 0 (0%) | 0 (0%) | 1 (2%) | 0 (0%) | 0 (0%) | 0 (0%) | 0 (0%) | 0 (0%) | 1 (1%) |
| **P06** Sleeplessness | 0 (0%) | 1 (2%) | 0 (0%) | 0 (0%) | 0 (0%) | 0 (0%) | 0 (0%) | 0 (0%) | 0 (0%) | 1 (1%) |
| **P12** Enuresis | 1 (2%) | 1 (2%) | 1 (2%) | 0 (0%) | 0 (0%) | 0 (0%) | 0 (0%) | 0 (0%) | 2 (1,6%) | 1 (1%) |
| **P13** Encopresis | 0 (0%) | 0 (0%) | 0 (0%) | 1 (2%) | 0 (0%) | 0 (0%) | 0 (0%) | 0 (0%) | 0 (0%) | 1 (1%) |
| **P15/P18/P19** Substance abuse | 0 (0%) | 0 (0%) | 0 (0%) | 1 (2%) | 0 (0%) | 1 (3%) | 0 (0%) | 0 (0%) | 0 (0%) | 2 (2%) |
| **P20** Memory/ concentration/ impairment | 0 (0%) | 0 (0%) | 0 (0%) | 0 (0%) | 0 (0%) | 2 (6%) | 0 (0%) | 0 (0%) | 0 (0%) | 2 (2%) |
| **P21** Hyperactivity/ hyperkinetic | 0 (0%) | 1 (2%) | 0 (0%) | 0 (0%) | 0 (0%) | 3 (9%) | 0 (0%) | 0 (0%) | 0 (0%) | 4 (3%) |
| **P22** Other worries behaviour child | 0 (0%) | 1 (2%) | 0 (0%) | 1 (2%) | 0 (0%) | 0 (0%) | 0 (0%) | 0 (0%) | 0 (0%) | 2 (2%) |
| **P24** learning problem | 0 (0%) | 0 (0%) | 0 (0%) | 1 (2%) | 0 (0%) | 0 (0%) | 1 (25%) | 0 (0%) | 1 (1%) | 1 (1%) |
| **P24.01** | 0 (0%) | 0 (0%) | 0 (0%) | 1 (2%) | 0 (0%) | 0 (0%) | 0 (0%) | 0 (0%) | 0 (0%) | 1 (1%) |
| **P24.03** Motoric development disorder | 0 (0%) | 1 (1%) | 0 (0%) | 0 (0%) | 0 (0%) | 0 (0%) | 0 (0%) | 0 (0%) | 0 (0%) | 1 (1%) |
| **P24.2** Language/ speech deficiency | 7 (14%) | 1 (2%) | 4 (9%) | 1 (2%) | 4 (13%) | 0 (0%) | 0 (0%) | 0 (0%) | 15 (12%) | 2 (2%) |
| **P29** Other psych. complaints | 0 (0%) | 1 (2%) | 0 (0%) | 0 (0%) | 0 (0%) | 1 (3.1%) | 0 (0%) | 0 (0%) | 0 (0%) | 2 (2%) |
| **P74** Anxietydisorder | 0 (0%) | 0 (0%) | 0 (0%) | 2 (5%) | 1 (3%) | 0 (0%) | 0 (0%) | 0 (0%) | 1 (1%) | 2 (2%) |
| **P78** Surmenage | 0 (0%) | 0 (0%) | 0 (0%) | 1 (2%) | 0 (0%) | 0 (0%) | 0 (0%) | 0 (0%) | 0 (0%) | 1 (1%) |
| **P99** Autism isorder | 0 (0%) | 1 (2%) | 0 (0%) | 1 (2%) | 1 (3%) | 0 (0%) | 0 (0%) | 0 (0%) | 1 (1%) | 2 (1%) |
| **Total** | 8 | 9 | 6 | 11 | 7 | 7 | 1 | 0 | 22 | 27 |

*Given the limited total number, percentages are rounded off to whole numbers
